# Systematic Evaluation of a Novel Self-Healing Poly(acrylamide-co-vinyl acetate)/Alginate Polymer Gel for Fluid Flow Control in High Temperature and High Salinity Reservoirs

**DOI:** 10.3390/polym13213616

**Published:** 2021-10-20

**Authors:** Jingyang Pu, Baojun Bai, Thomas P. Schuman

**Affiliations:** 1 School of Petroleum Engineering, China University of Petroleum Huadong, Qingdao 266580, China; 20200099@upc.edu.cn; 2 Department of Geosciences and Geological and Petroleum Engineering, Missouri University of Science and Technology, Rolla, MO 65409, USA; tschuman@mst.edu

**Keywords:** Alg-IPNG particle, self-healing, reinforcement, chromium acetate, Ca–alginate bond

## Abstract

Preferential fluid flow often occurs when water and CO_2_ is injected into mature oilfields, significantly reducing their injection efficiency. Particle gels have been evaluated and applied to control the short circulation problems. This study systematically investigated a novel poly(acrylamide-co-vinyl acetate)/alginate-based interpenetrated gel system (Alg-IPNG) which is designed to control the preferential fluid flow problems in high-temperature reservoirs. Chromium acetate was incorporated into the gel system to provide the delayed crosslinking feature of the particle gels. The alginate polymer system can also take advantage of the Ca^2+^ ions in the formation water, which exist in most reservoirs, to reinforce its strength by capturing the Ca^2+^ to form Ca–alginate bonds. In this paper, various characterizations for the Alg-IPNGs before and after the self-healing process were introduced: (1) the elastic modulus is set at up to 1890 Pa, and (2) the water uptake ratio is set at up to 20. In addition, we also discuss their possible self-healing and reinforcement mechanisms. In particular, the self-healing starting time of the Alg-IPNG particles are modified between 38 to 60 h, which is related to the water uptake ratio, Ca^2+^ concentration, and temperature. The reinforced Alg-IPNG gel has an enhanced thermal stability (180 days) at the temperature up to 110 °C.

## 1. Introduction

Hydrophilic polymeric gels, also known as superabsorbent polymers (SAPs), have attracted broad interest in medicine, drug delivery, drug release, enhanced oil recovery, and so on [1,2]. Dried SAPs can absorb several to even hundreds of times their mass of water to become swollen gels with a three-dimensional (3D) structure. The swelling ratio depends on their composition when it is synthesized and the environments that they are deployed in, such as salinity and ion type, pH, and temperature. 

SAPs, which have been often called preformed particle gels (PPG) in petroleum literatures, have been widely investigated and applied to control the preferential fluid flow in reservoirs and thus reduce water production [3]. Compared to their applications in other areas, the SAPs used in sub-surface fluid flow control are required to have controlled swelling ratios, excellent resistance to different ion compositions and reservoir temperatures, and sometimes delayed swelling rates. Extensive investigations have been conducted to modify the particle gel strength, swelling time, and thermostability, as well as their transport and plugging efficiency for various types of channels, such as fracture, void space conduits, and so on [4,5]. However, field applications and lab experiments have demonstrated that these conventional PPGs cannot control the reservoir conformance very well when they are applied in reservoirs with very severe channeling problems, such as large fractures, void space conduits, wormholes, and so on [6]. In such features, PPG particles could be flushed out from the production wells during subsequent water injection [7]. PPG plugging capabilities have been mentioned in a variety of studies and many approaches have been revealed, such as strength modification [8] and delayed swelling [9] by introducing multi-functional groups to the networks of the gel. However, these improvements cannot meet the blocking requirements of such large fracture-like features. The self-healing approach of functional hydrogels is considered as a solution for the current challenge in this paper.

The self-healing function [10], reported in many smart materials, has been considered as a promising way to improve the current preformed particle gel technology. For PPGs used in preferential fluid flow control, the self-healing property will make the swollen gel particles reassociate together to form a new robust bulk gel for a better plugging performance after the dispersed individual particles are placed in fractures or fracture-like channels. We have successfully developed a self-healable, partially hydrolyzed polyacrylamide-based (HPAM) polymeric hydrogel for the conformance control in low-temperature reservoirs [11]. However, the system cannot be applied in high-temperature and high-salinity conditions because its self-healing behavior was based on ionic bonds or coordinate bonds and its mechanical strength was not stable at high temperatures. High-temperature and high-salinity reservoirs are a familiar feature of oilfields and geothermal sites. It is of major importance when developing a novel self-healing polymer gel system that can be applied in such harsh reservoir conditions.

The polymeric incorporation (interpenetration) structure of multiple functional polymers has been proven as a method to toughen the current gel system for a high-temperature environment [12]. This method has initially been introduced by the development of an interpenetrating structure (IPN) of hydrogels [13] that consist of two or more intertwined brittle and ductile networks synthesized via a stepwise, sequential, free-radical polymerization [14]. Alginate is one of the more widely used natural ingredients for drug delivery through IPNs using natural polysaccharides. Alginate is hydrocolloid in the form of a linear copolymer of linked α-L-gulopyranuronic acid (G monosaccharides) and β-D-mannopyranuronic acid (M monosaccharides) [15]. Alginate was selected due to its inherent capability for ionic crosslinking through high transient calcium–carboxyl complexation, which allows for reversible energy dissipation by dissociation/reassociation [16]. In food applications, alginate can form a gel matrix in the presence of Ca^2+^, which can bind to four repeat units of G monosaccharide sequences to create a tough gel network. Therefore, when used as a plugging agent in reservoirs, the alginate polymer can help improve the gel strength, resulting in toughing the whole IPN structure and improving the salinity resistance.

In this study, a new Alg-IPNG particle gel was synthesized by forming the IPN structure of alginate and poly(acrylamide-co-vinyl acetate) (poly(AM-co-VAc)). The Alg-IPNG particle was shown to absorb water and swell when immersed in brine and was self-reinforced through the reactions of Ca^2+^ with four alginate G monosaccharide units. Entrapped chromium acetates contributed to a delayed self-healing reaction among the Alg-IPNG particles. The alginate also showed the excellent capability of adsorbing chromium acetates which can prevent chromium acetates from diffusing outside of the particle [17]. The swollen Alg-IPNG particle demonstrated its self-healing properties through the delayed replacement reaction between chromium acetate complexes and the carboxylic group of poly(AM-co-VAc). The water uptake behaviors, rheological properties, and the Ca–alginate bond property of the Alg-IPNG gels have been investigated. The self-healing capability and mechanical strength were characterized at different water uptake ratios and temperatures. The self-healed Alg-IPNG gel demonstrated high thermal stability up to 110 °C.

## 2. Experimental 

### 2.1. Materials

Sodium alginate (MW~90 kDa) was purchased from Acros Organics. Acrylamide (AM), vinyl acetate (VAc), and chromium acetate (${\left({\mathrm{CH}}_{3}\mathrm{COO}\right)}_{7}{\left(\mathrm{OH}\right)}_{2}{\mathrm{Cr}}_{3}$, Cr^3+^ ~24%) were obtained from Alfa Aesar. N,N’-methylene bisacrylamide (MBAA) was obtained from Sigma Aldrich at St. Louis, MO, USA. Ammonium persulfate (APS) were purchased from Fisher Scientific. All reagents were used as received. 

### 2.2. Preparation of Alg-IPNG Particles and Structural Study

The Alg-IPNG hydrogel was prepared through free-radical polymerization approach [18,19] of AM and VAc. For a typical polymerization, 12.9 g AM, 3.24 g VAc, 4.4 mg MBAA, and 0.32 g chromium acetate were mixed with 30 mL distilled water. The solution was mixed with 10 mL 1 wt% alginate, after which the mixture was stirred for 30 min with argon protection. The initiator APS (0.025 g) was added to the mixture to initiate the polymerization reaction at 50 °C. 

The formed hydrogel was cut and dried at 80 °C for 48 h, followed by a smash and screening process. The processed Alg-IPNG particles were cut and screened again in a variety of sizes for further characterization and evaluations. The Alg-IPNG with less than 96 µm (160 meshes) was used for FT-IR measurement. FT-IR measurement was used to identify the carboxylic group and the polysaccharide structures in the Alg-IPNG. The FT-IR samples were prepared using the KBr pellet method, and their spectra were recorded on a Thermo Nicolet Nexus 470 spectrometer (Thermo Scientific Inc., Shanghai, China) and processed by the OMNIC software. The morphology of swollen Alg-IPNG gels was characterized using a scanning electron microscope (SEM, Hitachi S4700, Tokyo, Japan). 

The stability of Cr^3+^ entrapment in the Alg-IPNG gels was characterized by a membrane dialysis experiment [20]. In the equilibrium dialysis experiment, 1 g of Alg-IPNG particles were put in a membrane bag (about 40 mL volume) made by a Spectra/Por Standard RC Tubing with two closures. The bag was filled with 0.1% CaCl_2_ before being immersed in 100 mL of 0.1% CaCl_2_ to allow ionic exchanges between the Ag-N mixture and the dialysate [21]. The Cr^3+^ concentration of the dialysate was measured using a Perkin-Elmer Model 3110 atomic absorption spectrophotometer (AAS) with a 359 nm laser light at 23 °C and used for calculating the accumulative dialyzed mass of the Cr^3+^. The mass of the released Cr^3+^ was calculated by integrating the Cr^3+^ concentrations of the dialysate. The entrapment efficiency of the Cr^3+^ is calculated as shown in Equation (1):

(1) $$\mathrm{Entrapment}\;\mathrm{efficiency}=\frac{M_{\mathrm{Cr},\mathrm{feed}}-M_{\mathrm{Cr},\mathrm{dialyzed}}}{M_{\mathrm{Cr},\mathrm{feed}}}\times 100\%$$

where $M_{\mathrm{Cr},\mathrm{feed}}$ represents the total feed Cr^3+^ (1 g) and $M_{\mathrm{Cr},\mathrm{dialyzed}}$ represents the total dialyzed Cr^3+^ after the dialysis experiment is finished.

The swelling rate and swelling equilibrium were determined by investigating the water uptake capability of the Alg-IPNG particles at room temperature (23 °C). Alg-IPNG particles were immersed in 50 mL solutions with a variety of brine types and salinity levels. For example, the study was performed by immersing the dry Alg-IPNG particles (0.5 g) in a 1 wt% CaCl_2_ solution (40 mL, assuming brine density is equal to 1 g/cm^3^) in a 50 mL transparent tube with scales. The water uptake behavior of the Alg-IPNG was investigated by reading the scales of the total solid volume. The water uptake ratio of the gel particles is calculated based on their mass in a swollen state (W_t_) divided by their mass in the dried state (W_0_), as shown in Equation (2):

(2) $$\mathrm{Water}\;\mathrm{uptake}\;\mathrm{ratio}=W_{t}/W_{0}$$

The equilibrium water uptake ratio of the Alg-IPNG particles was obtained if no further volume changed five hours. Half-swollen and full-swollen Alg-IPNGs were used in the evaluation.

### 2.3. Self-Healing and Rheological Study

The self-healing capability of both original hydrogel and swollen Alg-IPNG particles was investigated. To examine the self-healing capacity of the original hydrogel, one piece of freshly cracked Alg-IPNG hydrogel was put in contact with another piece of fresh hydrogel in the air. The cracked Alg-IPNG hydrogel was also in contact with IPNG (with no Cr^3+^) to prove the intermolecular crosslinking capability of Cr^3+^. The bottle test method [22] was applied to investigate the self-healing behavior of the Alg-IPNG particles after swelling. The swelled Alg-IPNG particles were aged at 65 °C, 80 °C, and 110 °C, with a controlled water uptake ratio varying from 10:1 to 40:1. Since the self-healing process was designed to be delayed, and healing is not an instantaneous process, we defined the self-healing times as the starting time, completion time, and time interval, same as in our previous publications [23]. The starting time was the time when some weak associations among the particles formed. In particular, the particles became tacked and some drawbenches were observed on the surface of the swollen particles. The completion time was the time when the particle boundaries disappeared, which meant a bulk gel has been formed and no individual particles could be recognized anymore. The self-healing time interval was the duration from the starting time to the completion time.

The mechanical strength of the equilibrium swollen Alg-IPNG particles was measured by measuring the elastic modulus (G’) using a Hakker MARS III rheometer (23 °C). The spindle used for the measurements was a parallel-plate geometry (PP35L Ti L) with a gap of 1 mm. The mechanical strength of the self-healed Alg-IPNG hydrogel was investigated using the same geometry by measuring the hydrogel cylinder with a thickness of 1.5 mm and a diameter of 2 cm at room temperature (23 °C). The measurement was set as an oscillation time-dependent experiment model at a fixed frequency of 1 Hz and with a controlled strain of 0.1% to perform shear elastic modulus as a function of time. 

### 2.4. Thermal Stability Evaluation

A thermal stability test was carried out by monitoring the volume and morphology changes of the self-healed Alg-IPNG gels under a variety of temperatures [24]. In detail, the dry Alg-IPNG particles were immersed in the 0.1% CaCl_2_ brine with a controlled volume to allow the particles to swell to the designed swelling ratio (10 to 20 times) before being moved into ampoules. A vacuum pump was run for approximately half an hour to remove the dissolved gases in the liquid samples, including any trace of dissolved oxygen that might have remained in the samples. Next, the ampoules were flame-sealed in place and aged at 110 °C.

## 3. Results and Discussions

### 3.1. Synthesis of Alg-IPNG Gels

**Figure 1:**
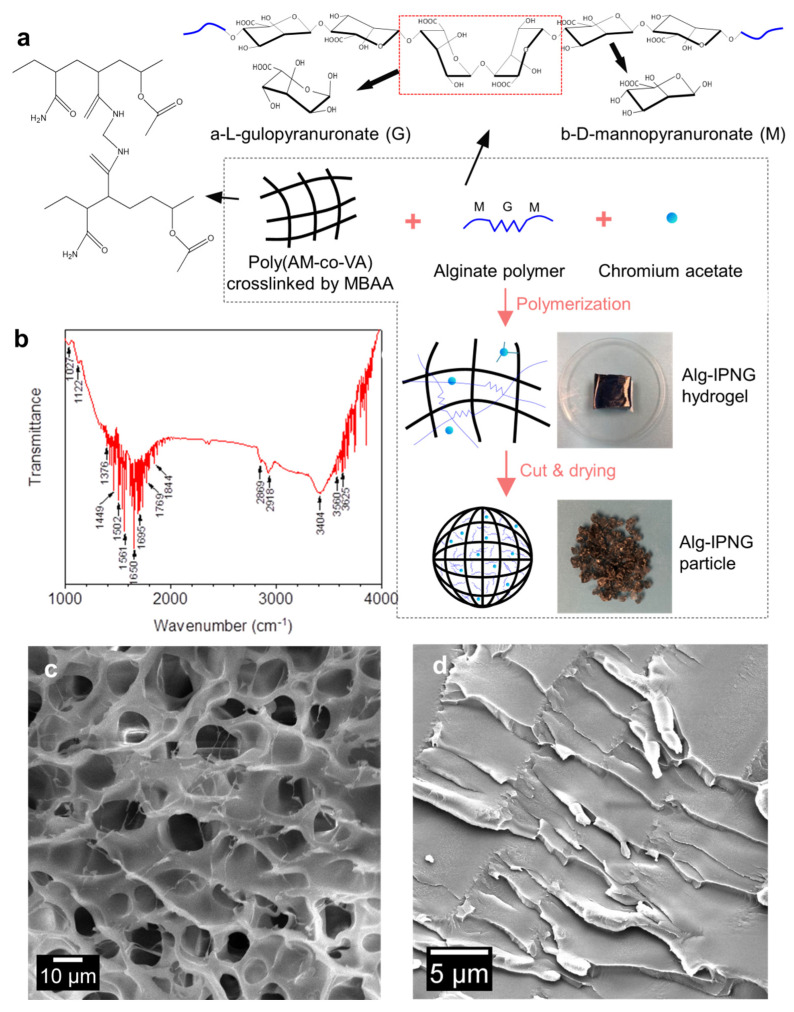
Schematic illustration of the preparation and the interpenetrating structure of the Alg−IPNG particle: (**a**) preparation; (**b**) FTIR detection result of the Alg−IPNG particles; (**c**,**d**) Microstructures of Alg-IPNG hydrogel and particle.

As shown in Figure 1, poly(AM-co-VAc) polymers were crosslinked by covalent bonds (MBAA) with interpenetrated alginate polymers. Poly(vinyl acetate), or PVAc, a leathery and water-resistant polymer, was chosen to improve the toughness of polyacrylamide [25]. Trivalent chromium (Cr^3+^) was considered to be responsible for the delayed interaction (self-healing) between the chromium acetate complexes and the carboxylic groups of the poly(AM-co-VAc) [26].The introduction of the Cr^3+^ acetate complexes reduced the rate at which the Cr-carboxylic group bonds were formed, which prolonged the reaction rate between the Cr complexes and the carboxylic groups on the poly(AM-co-VAc) [27]. The mechanism of the copolymerization of PVAc onto PAM, including the adsorption of the copolymer to chromium acetate, has been described in a previous article [28]. As a result of this process, a known amount of free-state Cr^3+^ was sealed in the Alg-IPNG gel. The alginate polymer was a linear copolymer of α-L-gulopyranuronate (G monosaccharides) and β-D-mannopyranuronate (M monosaccharides). The poly(AM-co-VAc) polymer and the alginate polymer formed an interpenetration structure with entrapped Cr complexes. The polysaccharide functional groups in the Alg-IPNG gel were identified using the FT-IR spectra to determine the existence of the alginate (Figure 1b). The absorption peaks at 1027 and 1122 cm^−1^ can be attributed to the ether group (-COC-) of saccharides. The peaks across 1550 to 1750 cm^−1^ can be attributed to the carboxylic acid group (-COOH) on the alginate and amide group (-CONH2) on the polyacrylamide. The swollen Alg-IPNG particle showed a typical porous continuous poly(AM-co-VAc) network with pore sizes smaller than 15 μm (measure using ImgeJ softwae). Many thin alginate polymers were entangled and interpenetrated within the poly(AM-co-VAc) polymers, which could enhance the particle’s mechanical strength (Figure 1c). The network structure was condensed after drying the Alg-IPNG hydrogel to form the Alg-IPNG particles, as shown in Figure 1d.

### 3.2. Water Uptake and Reinforcement of the Alg-IPNG Gel Particles

**Figure 2:**
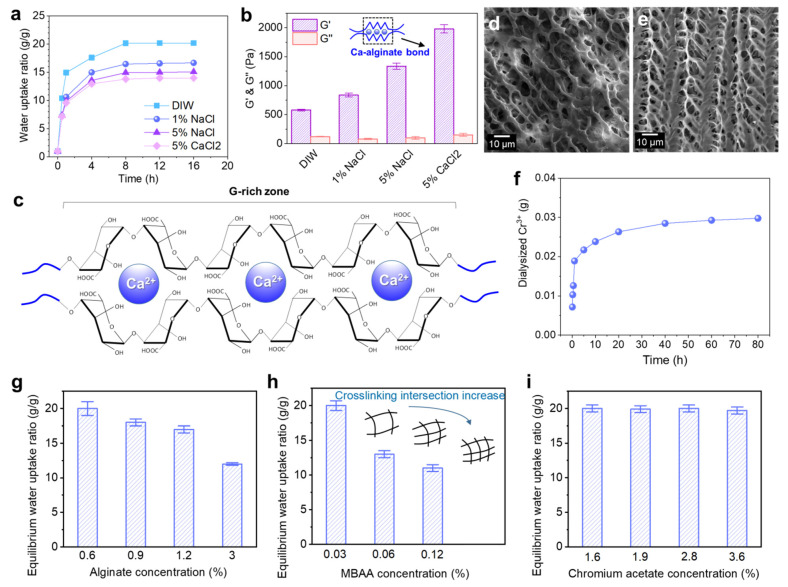
Water uptake and ionic exchange of the Alg-IPNG gels: (**a**) water uptake kinetic curves of the Alg-IPNG particles in different types of brines; (**b**) elastic modulus (G’) and loss modulus (G”) of the equilibrium swollen Alg-IPNG particles; (**c**) structure of the Ca–alginate bond; (**d**,**e**) micro-structures of the swollen Alg-IPNG particles immersed in 5% NaCl and 5% CaCl_2_. respectively. (**f**) The amount of dialyzed Cr^3+^ as a function of time; The effect of (**g**) alginate, (**h**) MBAA, and (**i**) chromium acetate on the swelling profile of the Alg-IPNG particles in 0.1% CaCl_2_.

The Alg-IPNG particle demonstrated both water uptake and ion-exchange properties. Figure 2a demonstrated the effect of brine concentration and type on the swelling properties of the Alg-IPNG particles. The result showed that the water uptake kinetic or equilibrium of the Alg-IPNG was reduced as the brine Na^+^ concentration increased. For example, the equilibrium water uptake ratio of the Alg-IPNG was 20 in DIW and 14 in 5% CaCl_2_ brine.

Figure 2b demonstrates the effect of brine types on the rheological properties of Alg-IPNG. It was found that the elastic module G’ of the fully swollen Alg-IPNG increased with Na^+^ concentrations, since the carboxylate of poly(AM-co-Vac) was compressed by Na^+^, resulting in a higher elastic modulus (1335 Pa) than in DIW (580 Pa, Figure 2c). In addition, the G’ of the fully swollen Alg-IPNG particles that were immersed in 5% CaCl_2_ brine (1890 Pa) was higher than that of those immersed in 5% NaCl brine. The results relating to the equilibrium swelling ratio revealed that Ca^2+^ enforced the swelled Alg-IPNG particles. Other cations, such as Na^+^ and Cr^3+^, would not react with alginate, while Ca^2+^ would bond with alginate to form Ca–alginate bonds. Therefore, the Ca–alginate bond improved the elastic modulus of the Alg-IPNG gels, which is supported by previous alginate studies [29]. The G’’ of the equilibrium-swollen Alg-IPNG did not change very much with the variety of the solutions (115 ± 35 Pa). When comparing the SEM microstructures of the fully swollen Alg-IPNG gels with 5% NaCl (Figure 2d) and 5% CaCl_2_ (Figure 2e), we found that the latter one demonstrated more taut and regular network structures than the former one, which proved the existence of the Ca–alginate bond.

Furthermore, the dialysis experiment of the Alg-IPNG in 0.1% CaCl_2_ was used to study the entrapment efficiency of the Cr^3+^ in the swollen Alg-IPNG particles. As shown in Figure 2f, the total dialyzed amount of Cr^3+^ from the Alg-IPNG particles increased significantly with the aging time in the first 2 h and moved slowly toward equilibrium after 40 h. The total dialyzed amount of Cr^3+^ was 2.88 mg, leaving a majority of Cr^3+^ remaining in the Alg-IPNG particles. The high entrapment efficiency (78.5%) of Cr^3+^ in the Alg-IPNG particles was due to the cooperation of the adsorption capabilities of the carboxylic groups on the poly(AM-co-VA) to Cr^3+^, which has been proven in many previous reports [20,30].

The water uptake of the Alg-IPNG particles was controlled for by investigating the effect of the alginate concentration, MBAA concentration, and chromium acetate concentration on the equilibrium swelling ratio of the Alg-IPNG particles. The samples were immersed in a low-concentration CaCl_2_ solution (0.1%) for at least 48 h for particle swelling. The results showed that the equilibrium water uptake ratio decreased greatly from 20 to 12 with the increase in the alginate concentration (Figure 2g). It was known that the acidic form of alginate was not water soluble, but its sodium salt was [31]. When immersed in a calcium solution, the alginate became water insoluble slowly with the loss of sodium ions, resulting in a swelling reduction. Moreover, excess alginate polymer reduced the integrity of the crosslinked poly(AM-co-VAc) egg-box structures, inhibiting its swelling capability. The MBAA crosslinker concentration directly influenced the equilibrium water uptake ratio of the Alg-IPNG particles, reducing the ratio from 20 to 11.5 as the MBAA concentration increased from 0.03% to 0.12% (Figure 2h). The increased crosslinking intersection of the poly(AM-co-VAc) network increased the stiffnesses of the Alg-IPNG particles, while at the same time reducing the water uptake capability. The concentration of Cr^3+^ had no noticeable effects on the Alg-IPNG particle-swelling kinetics, and the equilibrium water uptake ratio of the Alg-IPNG particles stayed the same as the chromium acetate concentration increased from 1.6% to 3.6% (Figure 2i). This result was in agreement with our previous self-healing hydrogel technology study [28], which demonstrated the chromium acetate entrapment capability of the polyacrylic-based hydrogels. Most chromium acetates did not participate in forming the network structures during the Alg-IPNG preparation and did not increase the crosslinking intersection of the poly(AM-co-VAc) polymers.

### 3.3. Self-Healing Behavior of Swollen Alg-IPNG Particle

**Figure 3:**
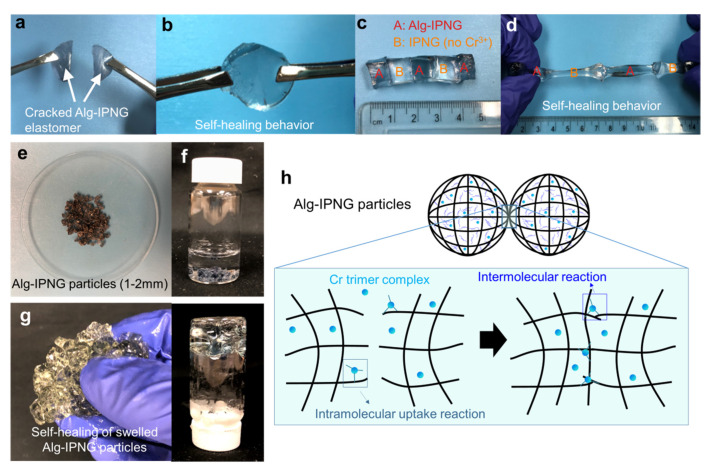
Self-healing of the Alg-IPNG gel: (**a**,**b**) self-healing of a broken Alg-IPNG gel; (**c**,**d**) self-healing between Alg-IPNG and IPNG gels; (**e**–**g**) photograph of the Alg-IPNG particles; (**h**) schematic illustration of the self-healing process of the Alg-IPNG gel—delayed self-healing by chromium trimer complexes at the contact interfaces of the gel.

The self-healing behaviors of both wet hydrogels and swollen Alg-IPNG particles were investigated as shown in Figure 3. In detail, two freshly cracked large pieces of Alg-IPNG gels (Figure 3a) were put together in contact with each other, and were aged at 65 °C without adding any healing agent. It can be clearly seen that the Alg-IPNG hydrogels self-healed with each other at the contact interface without a visible joint interface after 48 h (Figure 3b). The self-healed gel can further withstand gentle stretching. Additionally, the self-healing has been shown to occur between cracked Alg-IPNG hydrogel and IPNG (Alg-IPNG without Cr^3+^) hydrogel (Figure 3c), while the self-healed gel has been shown to have elastomer properties, being able to stretch to more than two times its original length (Figure 3d). As the self-healed gel was stretched, a necking phenomenon occurred at one piece of the gels, but not at the two contact interfaces, while the self-healing did not fail by separating the gel pieces at the contacted interfaces. The appearance of the dry Alg-IPNG particles was shown in Figure 3e. After being immersed in 1% NaCl brine (Figure 3f) for 48 h at 65 °C, the swollen Alg-IPNG autonomously reformed into a bulky gel (Figure 3g). The self-healed bulky gel was stable in the liquid environment.

Enhanced self-healing performance and excellent rheological strength are usually regarded as contradictory properties of hydrogels. A conventional hydrogel could not heal itself mostly due to the irreversible and permanent breakage of the covalent crosslinks [32,33]. In this study, the bonds between Cr ions and carboxylic groups were introduced in our study, which was known as dynamic reversible bonds which performed a delayed-bonding reaction after a predetermined time with the introduction of the acetate molecules [7]. The delayed self-healing mechanism of the Alg-IPNG gel was inspired by the known chromium acetate-HPAM gel system, in which the chromium formed complex ions in a solution and reacted via a replacement reaction with the carboxylic on the polymer molecules to form bonds resulting in a new network or gel. The replacement reactions are divided into two steps, uptake reaction (Equation (3)), and replacement reaction (Equation (4)):

Uptake reaction:

Cr(Ac^−^)_3_ + mP1-CO_2_^−^ → Cr(L)_3-m_(P1-CO_2_^−^)_m_ + m(Ac^−^) (3)

Replacement reaction:

Cr(Ac^−^)_3-m_(P1-CO_2_^−^)_m_ + nP2-CO_2_^−^ → (P2-CO_2_^−^)_n_Cr(Ac^−^)_3-m-n_(P1-CO_2_^−^)_m_ + (m + n)(Ac^−^) (4)

where Ac^−^ represents an acetate ligand in the chromium acetate and -CO_2_^−^ represents a carboxylic group on a polymer molecule (P1 or P2). Tackett [27] found that at pH values below 4.5, a green cyclic chromium trimer was the dominant species in the solution. The chromium trimer was not stable. As the pH of the solution is increased, hydroxyl groups replace the bridging acetate group. The instability of the chromium trimer leads to the self-healing capability of the Alg-IPNG hydrogels and the swollen Alg-IPNG particles. Without adding any extra crosslinking agents, the spontaneous reaction between Cr trimers and carboxylic groups was formed at the point of contact between the Alg-IPNG gels (Figure 3h). The self-healing reaction was controllable on time and completed without a visible joint interface. Therefore, the interpenetrating structure of two polymers in the self-healed Alg-IPNG hydrogel was still homogenously distributed.

**Figure 4:**
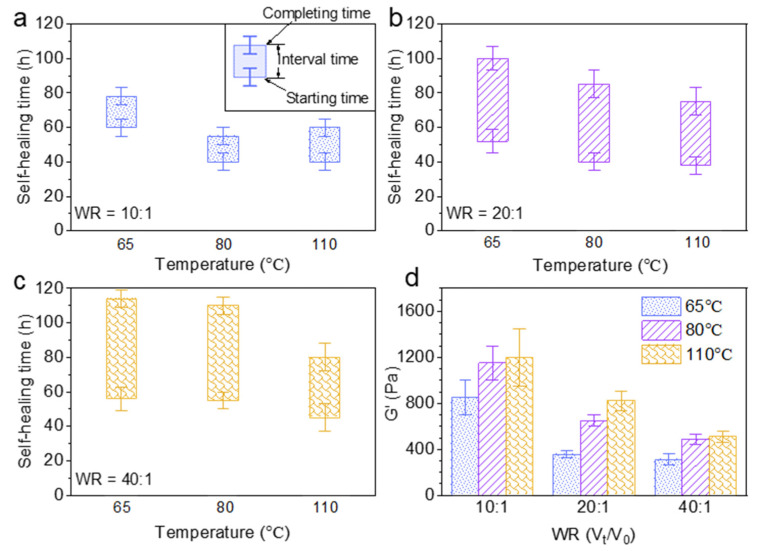
Self-healing time study of Alg-IPNG particles when: (**a**) half swollen; (**b**) fully swollen; (**c**) fully swollen with excess brine; and (**d**) the elastic modulus (G’) of the healed gels.

The effect of the water uptake ratio (V_t_/V_0_) and temperature on the self-healing time and rheological property of Alg-IPNG particles was investigated. The brine amount was controlled to render the Alg-IPNG particles half-swollen (10:1), fully swollen (20:1), and fully swollen with excess brine (40:1). Figure 4a showed that the starting time of the Alg-IPNG gels was decreased from 60 to 40 h with the increase in the temperature from 65 °C to 110 °C. For the samples at higher water uptake ratios, the starting time was reduced further with the increase in temperatures (from 52 to 38 at 80 °C and from 71 to 45 at 110 °C; Figure 4b,c). On the other hand, the water uptake ratio of the Alg-IPNG particles had a strong effect on the self-healing interval time, compared with the temperature. For example, the interval time increased from 18 to 49 h when the water uptake ratio increased from 10:1 to 40:1 at 65 °C. The increase in the interval time showed similar trends at 80 °C and 110 °C. Additionally, the interval time reduction trend was not linear as the temperature increased. Figure 4d illustrates the effect of the water uptake ratio and temperature on the G’ of the self-healed Alg-IPNG gel. For example, the G’ with the water uptake ratio at 10:1 was nearly two times that with the water uptake ratio at 20:1 or 40:1. The G’ increased with temperature. For example, as the temperature increased from 65 °C to 110 °C at the water uptake ratio at 10:1, the G’ increased from 850 to 1210 Pa. Interestingly, this result was opposite to the other previously performed particle gel experiments reported by us [7,34] or other researchers who showed relatively capable materials as the temperature increased to a high level, such as 110 °C.

**Figure 5:**
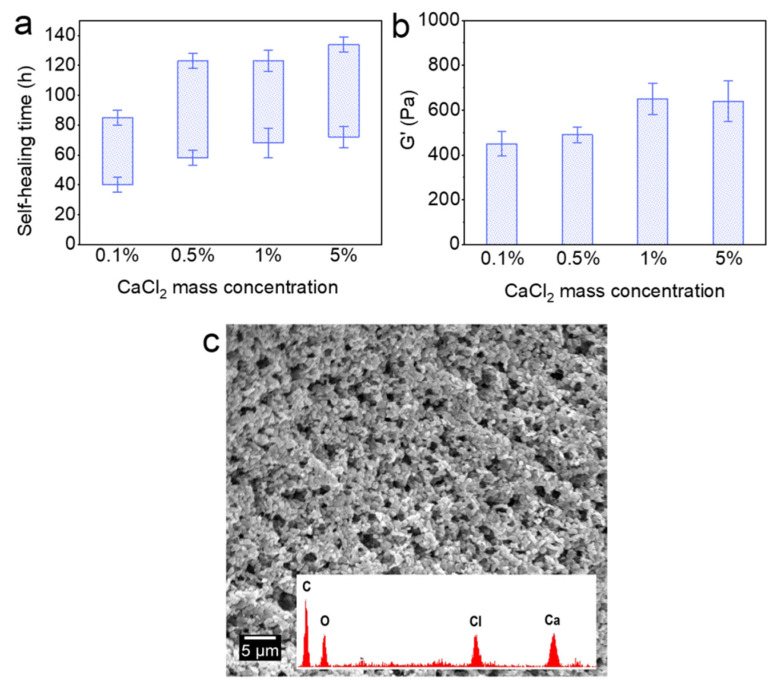
Self-healing of the Alg-IPNG gels at 65 °C: (**a**) self-healing time; (**b**) elastic modulus (G’) of the healed gel; and (**c**) micro-structure of the healed gel.

Figure 5 shows the effect of Ca^2+^ on the self-healing time and G’ of the Alg-IPNG particles. With the increase in the CaCl_2_ concentration, both the self-healing time and G’ of the self-healed Alg-IPNG gel were retarded. In detail, the starting self-healing time was delayed from 40 to 72 h as the CaCl_2_ mass percentage increased from 0.1 to 5%. Furthermore, the interval time was increased from 45 to 62 h for each of the samples (Figure 5a). As the CaCl_2_ concentration increased, the G’ of the Alg-IPNG gels increased and reached the highest G’ (650 Pa) in 1% CaCl_2_ brine. Before reaching 1%, an increase in Ca^2+^ concentration led to an increase in the mechanical strength of the Alg-IPNG by increasing the Ca–alginate bond number in the Alg-IPNG gel (Figure 5b). However, when the Ca^2+^ mass percentage reached a high concentration (5%), it hampered the self-healing in the Alg-IPNG gels. On one hand, a highly concentrated Ca–alginate bond on the alginate would limit the rearrangement, approach, and wetting of the poly(AM-co-VAc) and reduce the contact between the carboxylic groups and Cr^3+^. On the other hand, the surface of the Alg-IPNG gel was covered by the Ca–alginate bond which could reduce the contact interfaces of the Alg-IPNG gels (Figure 5c).

### 3.4. Thermal Stability of the Self-Healed Alg-IPNG

**Figure 6:**
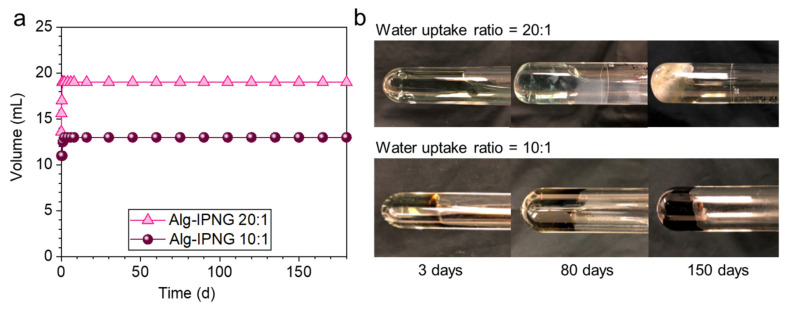
Thermal stability comparisons of the self-healed Alg-IPNG gels at 10:1 and 20:1 water uptake ratio at 110 °C in 0.1% CaCl_2_: (**a**) Volume changes as a function of aging time and (**b**) morphology changes in 150 days.

Self-healed Alg-IPNG bulky gels were aged at 110 °C to study their thermal stability by monitoring the volume and phase change. After being aged for 180 days, the Alg-IPNG gels remained stable regardless of the different water uptake ratios (Figure 6, left). From the results of the sample observations at different periods, it was found that the Alg-IPNG samples remained robust and stable for 180 days, during which the self-healed gel became a dark-green color with the water uptake ratio at 10:1 and a light-green color with the water uptake ratio at 20:1.

## 4. Conclusions

This study systematically developed and evaluated a chromium acetate-entrapped, self-healing Alg-IPNG preformed particle gel that can be used to plug high-temperature reservoirs that have fractures or fracture-like conduit problems. A stable network structure can be formed by the formation of MBAA-crosslinked poly(AM-co-VAc) and alginate polymer. The water uptake ratio of the Alg-IPNG particles ranges from 14 to 20 times their initial volume depending on the environment. The elastic modulus of the Alg-IPNG particles can reach up to 1890 Pa, which is greatly influenced by the water uptake ratio and brine type. Both the cracked Alg-IPNG hydrogel and the swollen Alg-IPNG particles are self-healing and able to reform bulky gels which are robust and stretchable. The self-healing property was attributed to the delayed reaction between the Cr^3+^ and the carboxylic group on the contact surfaces of different particles. The self-healing starting time of the Alg-IPNG particles can be controlled to be between 38 to 60 h to make sure it can be delivered into the reservoir before a bulk gel is formed. Ca^2+^ ion influenced the self-healing time and helped to reinforce the strength of the reformed bulk gel by forming Ca–alginate bonds and creating a hard shell on the gel surface. Both interpenetration structures of the Alg-IPNG and the Ca shell helped to increase the thermal stability of the self-healed robust gel at temperatures of up to 110 °C. We prospect that this approach can also promote further research in biochemistry for discovering novel self-healable SAP or SAPs with IPN structures.

## Data Availability

The data presented in this study are available on request from the corresponding author.
